# Enhanced attraction of sand fly vectors of *Leishmania infantum* to dogs infected with zoonotic visceral leishmaniasis

**DOI:** 10.1371/journal.pntd.0009647

**Published:** 2021-07-27

**Authors:** Ifhem Chelbi, Khouloud Maghraoui, Sami Zhioua, Saifedine Cherni, Imen Labidi, Abhay Satoskar, James G. C. Hamilton, Elyes Zhioua

**Affiliations:** 1 Unit of Vector Ecology, Institut Pasteur de Tunis, Tunis, Tunisia; 2 Laboratory of Bio-informatic, Mathematics, Statistic, Institut Pasteur de Tunis, Tunis, Tunisia; 3 Departments of Pathology and Microbiology, Ohio State University, Columbus, Ohio, United States of America; 4 Division of Biomedical and Life Sciences, Lancaster University, Lancaster, United Kingdom; Universidade Federal de Minas Gerais, BRAZIL

## Abstract

**Background:**

The sand fly *Phlebotomus perniciosus* is the main vector of *Leishmania infantum*, etiological agent of zoonotic visceral leishmaniasis in the Western Mediterranean basin. Dogs are the main reservoir host of this disease. The main objective of this study was to determine, under both laboratory and field conditions, if dogs infected with *L*. *infantum*, were more attractive to female *P*. *perniciosus* than uninfected dogs.

**Methodology/Principal findings:**

We carried out a series of host choice experiments and found that infected dogs were significantly more attractive to *P*. *perniciosus* than uninfected dogs in the laboratory as well as in the field. Significantly more *P*. *perniciosus* fed on infected dogs than on uninfected dogs. However, the fecundity of *P*. *perniciosus* fed on infected dogs was adversely impacted compared to uninfected dogs by lowering the number of laid eggs. *Phlebotomus perfiliewi*, the second most abundant sand fly species in the field site and a competent vector of *L*. *infantum* had similar trends of attractivity as *P*. *perniciosus* toward infected dogs under field conditions.

**Conclusions:**

The results strongly suggest that *L*. *infantum* causes physiological changes in the reservoir host which lead to the host becoming more attractive to both male and female *P*. *perniciosus*. These changes are likely to improve the chance of successful transmission because of increased contact with infected hosts and therefore, infected dogs should be particularly targeted in the control of zoonotic visceral leishmaniasis in North Africa.

## Author summary

Zoonotic visceral leishmaniasis is a neglected tropical disease caused by the parasite *Leishmania infantum*. In the Western Mediterranean basin, *Phlebotomus perniciosus* and *Phlebotomus perfiliewi* are the main vectors of *L*. *infantum*, and dogs are the main reservoir host of the parasite. In Northern Africa, ZVL affect mostly children less than 5 years. Understanding the chemical ecology governing the relationships between the vector, the parasite and the reservoir host is of major epidemiological importance. Previous studies based on rodent models have shown that the infection with *L*. *infantum* enhanced the attractiveness towards *Lutzomyia longipaplis*, the main vector of ZVL in the Americas. In this study, we reported for the first time that infected dogs are highly attractive to both male and female *P*. *perniciosus* under laboratory and filed conditions compared to uninfected dogs. Similar patterns were observed with wild populations of *P*. *perfiliewi*. Our results provided strong evidence that the parasite manipulate the reservoir hosts to enhance its transmission success by the vector.

## Introduction

Zoonotic visceral leishmaniasis (ZVL) is a vector-borne zoonotic disease caused by the parasite *Leishmania infantum*, which is transmitted by the bite of female phlebotomine sand flies. ZVL can affect both humans and canines and is considered by the WHO to be one of the most important neglected tropical diseases. It affects about 0.5 million people per year [1,2] and is widespread in South and Central America, North Africa, Southern Europe, Middle and Far eastern countries. ZVL distribution is strongly correlated with poverty [3] and in Tunisia is a peri-domestic disease mostly endemic in rural areas affecting families of low social and economic status [4].

ZVL is a systemic disease that frequentlyresults in the death of infected individuals if untreated. No effective vaccine for human visceral leishmaniasis is available [5] and in Tunisia, the disease has an estimated incidence between 100 and 160 cases per 100,000 inhabitants [2] with a mortality rate of 6% [6]. The incidence of ZVL is highest among children between 1 and 2 years of age [4]. In a previous study, we showed that paediatric patients admitted 15 days after onset of symptoms, with bleeding, white cell counts below 4,000/mm^3^, and cytolysis at admission should be considered severe cases and subsequently, they are at high risk of mortality [7].

Domestic dogs are the main reservoir host for *L*. *infantum* in the Old World [8,9] and in the New World [10] and sand flies of the subgenus *Larroussius*, predominantly *Phlebotomus perniciosus*, are the main vectors of ZVL in Tunisia [9,11,12]. Other sand fly species of the subgenus *Larroussius*, mainly *Phlebotomus perfiliewi*, also play an important role in the transmission of ZVL throughout the Mediterranean basin including Tunisia [12–14]. In previous studies in Tunisia we showed that the prevalence of ZVL in dogs is an important parameter for determining transmission to humans [4,15]. Therefore, understanding the dynamics of transmission of the parasite between the canine reservoir host and the sand fly vector *P*. *perniciosus* is of major epidemiological importance.

Sand flies use odours, heat, CO_2_ of the host [16–18], and sex/aggregation pheromones emitted by male sand flies [19,20] to identify and orientate towards potential host animals for blood-meal and mate acquisition. While *P*. *perniciosus* can feed on a wide variety of vertebrate hosts to obtain blood meals [21], dogs remain the main reservoir host for *L*. *infantum* [4,9]. From an eco-epidemiological view, the parasite requires an overlap between the vector (*P*. *perniciosus*) and the reservoir host (dogs), which is a prerequisite for the emergence of a ZVL focus. To achieve this overlap, some parasites are known to manipulate the host animal by changing its odour or behaviour to improve their chances of transmission [22]. It was shown that following infection with *L*. *infantum*, hamsters became significantly more attractive to females *Lutzomyia longipalpis*, vector of ZVL in South America [23,24].

We hypothesized that physiological changes in dogs parasitized by *L*. *infantum* change the dog’s odour making them more attractive to *P*. *perniciosus* and therefore enhancing the parasite’ s transmission success. To test this hypothesis, attractiveness of infected and uninfected dogs to *P*. *perniciosus* were assessed under both laboratory and field conditions.

## Materials and methods

### Ethics statement

The maintenance of animals and the experimental procedures used in this research program followed the Animal Care and Use Protocol which is approved by the Institutional Animal Care and Use Committee of the Institut Pasteur de Tunis, Tunisia (2018/01/I/ES/IPT/V0). Infected dogs used in this research program were obtained from a previous study that was approved by the Institutional Animal Care and Use Committee of the Institut Pasteur de Tunis, Tunisia (IPT/UESV/27/2012). This work was performed under the Assurance of the US Office of Laboratory Animal Welfare [Assurance approval F-16-00170 (A5743-01)]. The Institut Pasteur de Tunis complies with the European Directive for the Protection of Vertebrate Animals used for experimental and other scientific purposes (2010/63/EU).

### Assessing the attractiveness of female *Phlebotomus perniciosus* to infected and uninfected dogs under laboratory conditions

Sand flies used in this study were from a colony originated from Tunisia and maintained at the Laboratory of Vector Ecology in the Institut Pasteur de Tunis since 2003 [25,26]. Dogs used in the study were from the kennels of the Institut Pasteur de Tunis. We used 6 Beagle dogs that had been naturally infected with *L*. *infantum* as part of a different study to investigate the efficacy of a vaccine against canine ZVL. These dogs had been exposed to wild sand fly bites under natural conditions in a ZVL focus located at Borj Youssef in the governorate of Ariana (36°57’N, 10° 05’E) and were from the unvaccinated (control) group. Infection status of the infected dogs was confirmed by indirect immunofluorescent antibody test (IFAT) as described by Ben Slimane et al. (2014) [9]. All *Leishmania* species isolated from field-collected female *P*. *perniciosus* in Tunisia were identified as *L*. *infantum* zymodeme MON-1 [11,21]. In Tunisia, the zymodeme MON-1 is responsible for the majority of human and canine cases [8,27]. The dogs were kept in the kennels after the exposure period and showed clinical signs 11 to 14 months after being exposed to wild sand fly bites. All symptomatic dogs showing specific signs of ZVL including lymphadenomegaly, hepatomegaly, splenomegaly, and progressive weight loss were classified as infected when introduced into our study. In addition, 6 uninfected Beagles from the kennel were used as controls.

The infectiousness the infected and uninfected dogs to sand flies was confirmed by xenodiagnosis [9]. A minimum of 36% to 100% of the lab-colonised *P*. *perniciosus* that were fed on infected dogs developed *L*. *infantum* infections when dissected and examined under dissecting microscope as described by Chelbi and Zhioua (2019) [28], whereas none of the sand flies fed on uninfected dogs were found to be infected with parasites.

Infected dogs were housed individually in one part of the dog kennel where they received daily regular veterinary care. Uninfected dogs were housed in a separate part of the kennels. Protective measures were taken to avoid the infection of the uninfected dogs as described by Ben Slimane et al. (2014) [9]. Each infected dog was paired by sex (6 males and 6 females) and age (varying from 3 to 4 years old) but not weight with an uninfected one. These pairs were then used in both the laboratory and field experiments.

**Fig 1:**
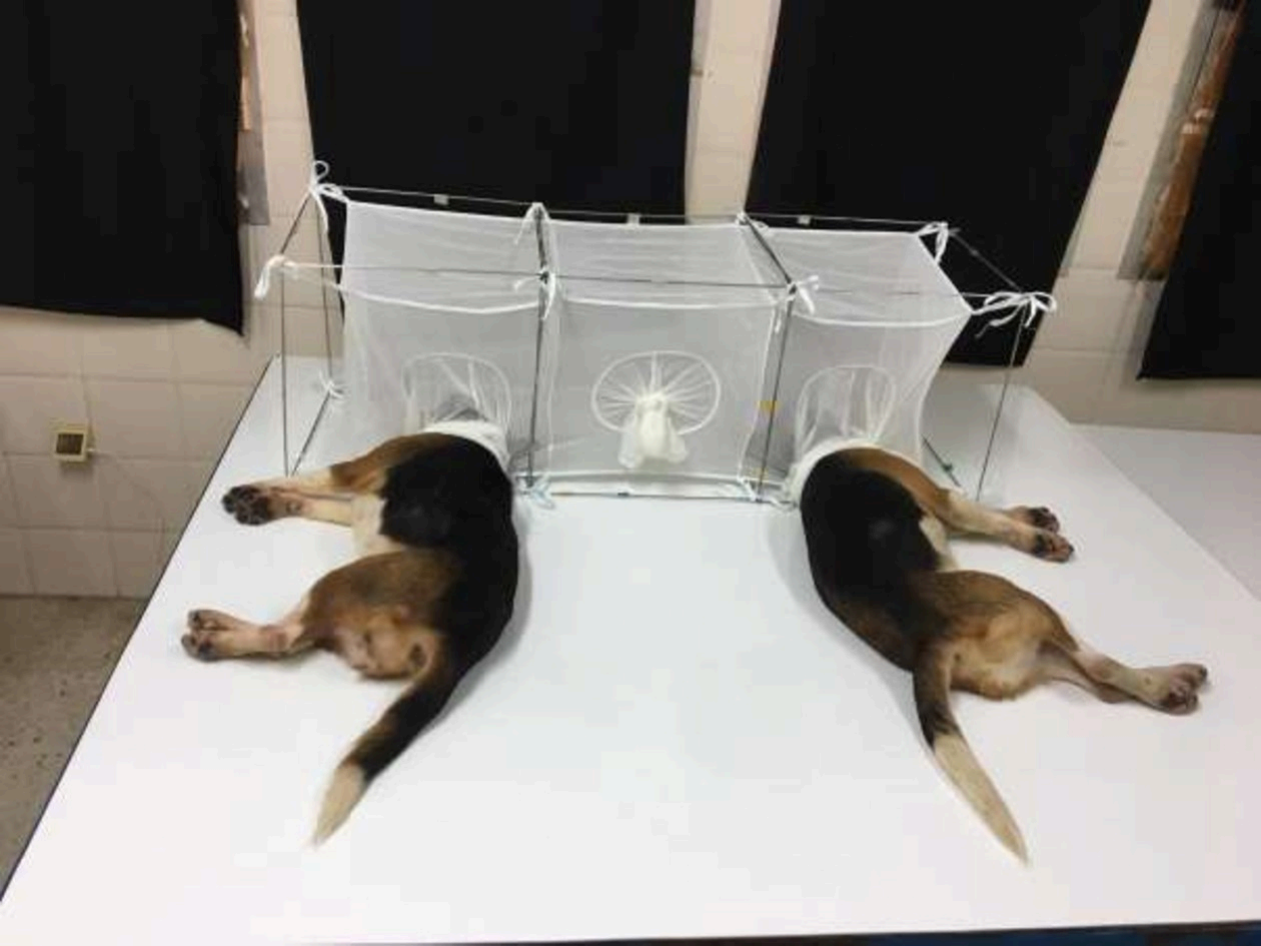
Host attractiveness experiment in the laboratory.

To assess the preference of female *P*. *perniciosus* for infected or uninfected dogs, we carried out a simple choice experiment (Fig 1). The wire frames of three Barraud cages (cage A, B, and C) each measuring (40 x 40 x 40 cm) were welded to each other to create a frame that was 120 x 40 x 40 cm. Three netting cages were suspended in a line within the frame. The middle netting cage (B) was connected to the two cages on the left (A) and right (C) through openings (diameter x 10 cm) cut in the netting. One infected dog and its’ uninfected pair were anaesthetized by intramuscular (IM) injection of a mixture of 1.5 ml of ketamine (15 mg/Kg) (Merial, Lyon, France) and 0.02 ml/Kg of acepromazine maleate (Kela, N.V., Hoogstraten, Belgium). As ear skin is the best predictor of being infectious to vectors [29], the head of the infected and uninfected dogs were placed in the sections A and C respectively of the three connected-cages for 80 mins (Fig 1). A minimum of 200 females and 20 males uninfected laboratory-colonized *P*. *perniciosus* (F 29) were then released into the central part of the cage (B) and the females given the opportunity to choose either the infected or uninfected host in section A or C. Male sand flies were present to encourage female feeding [25,26] but we did not determine their preferences. Unfed sand flies were 5 to 7 days old and deprived of sugar for 24 hours prior to use in this experiment.

Access from the central part B of the apparatus to sections A and C was initially restricted by netting placed over the connecting openings. After the sand flies had been introduced into the central section, they were allowed to acclimatize for (20 mins) then the netting covering the connecting openings to both sections A and C were removed to allow the free movement of *P*. *perniciosus* toward either the infected or uninfected dog. After 20 minutes, the connecting openings between the central section B and side sections A and C were closed. The sand flies were then allowed to feed on the sedated dogs for 60 min in the dark at 27°C. After this time the number of engorged and unfed female *P*. *perniciosus* in section A and C were counted as well as the number of females in section B [considered as not responding (NR)] were counted. The experiment was replicated six times with six different pairs of infected and uninfected dogs. For each pair of dogs we carried out 5 replicates. A total of 30 replicates were done for this experiment. To control for any effect side bias, the positions of the infected and uninfected dogs were swapped every replicate. The work was performed in a bio-safety level laboratory 2.

We compared the fecundity of sand flies fed on infected and uninfected dogs. Engorged females were held individually in glass vials (5 cm high and 2.5 cm diameter) containing a small wet filter paper, with access to cotton wool soaked in a sucrose solution (30%). Vials were maintained at 27°C and 90% relative humidity in sealed polythene containers. Flies were examined daily for up to 10 days post-blood feeding; those in the infected groups that had died the previous day were dissected and examined for the presence of parasites in the gut. Only those flies in which parasites were observed (N = 75) were considered to be infected and used in the analysis. For each female sand fly, the number of eggs laid plus those retained in the ovaries after death were recorded. As this experiment is extremely time consuming, we compared the fecundity of a subset of sand flies fed on two pairs of infected and uninfected dogs. Thus with the first pair of dogs we examined 47 females engorged on the infected dog and 42 on the uninfected dog and for the 2^nd^ pair of dogs 25 females engorged on the infected and 30 on the uninfected dog.

### Assessing the attractiveness of *Phlebotomus perniciosus* to infected and uninfected dogs under field conditions

This study was performed in an endemic area for ZVL where *P*. *perniciosus* is the most abundant sand fly species followed by *P*. *perfiliewi* [30]. The study took place during 9 consecutive nights in September 2019, a period corresponding to the main peak of activity of *P*. *perniciosus* and *P*. *perfiliewi* [31], at a rural dog kennel (36°58’N, 10°03’E) licensed by the Department of Agriculture and belonging to the Governorate of Ariana in northern Tunisia.

**Fig 2:**
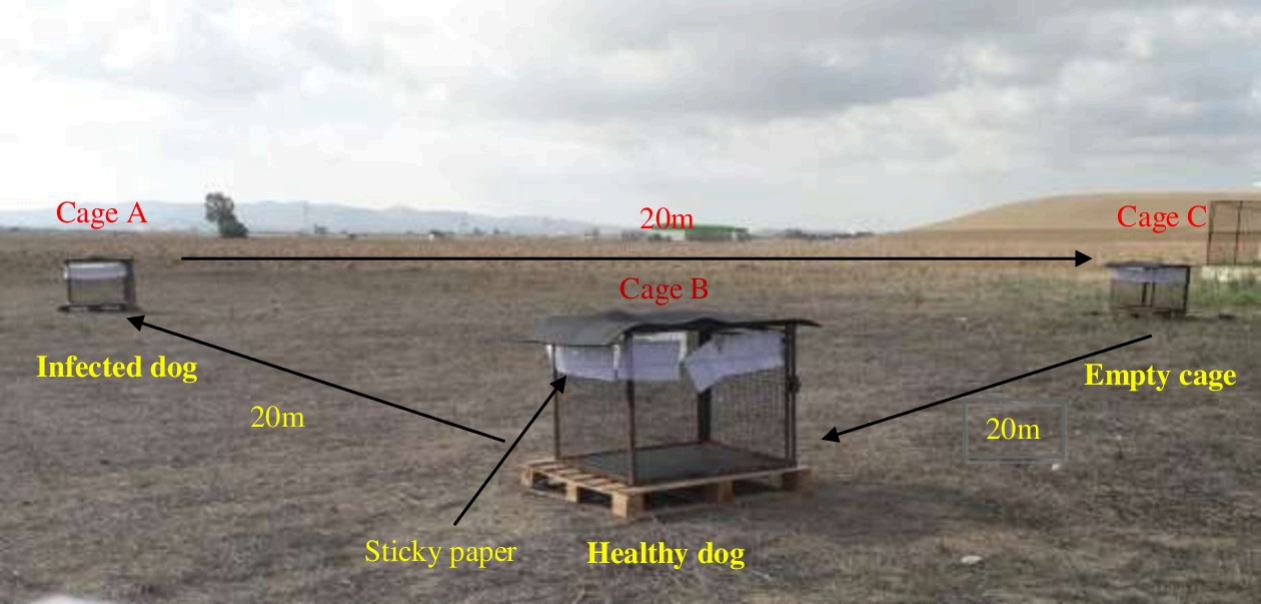
Host attractiveness experiment in the field.

An experiment was carried out to determine the preference of primarily wild *P*. *perniciosus* and secondly of *P*. *perfiliewi* for either infected (symptomatic) or uninfected (asymptomatic) dogs. Three cages (100 × 90 × 100 cm) were placed on the ground, in a triangular pattern, equidistant (20 m) from each other (Fig 2). We used one pair of dogs that we had used in the previous laboratory experiments. In one cage, we placed an infected dog and in the 2^nd^ we placed the uninfected paired dog. The 3^rd^ cage was left empty as a negative control. Sand flies were collected at the cages using sticky traps made with, 13 white papers (20 cm x 20 cm; total area 1 m^2^) soaked in castor oil. The sticky traps were attached 1 m above the ground and evenly spaced along a cord around the top of the cage (Fig 2). The density is reported as the number of sand flies of each species per 1 m^2^ of sticky traps.

The animals were tested, between 18:00–06:00 HR and received water *ad libitum* during the night. The experiment was replicated 9 times and the cages were rotated between different positions to avoid positional bias. Sand flies collected using sticky traps were counted and identified to species level by using identification keys [32,33]. The atypical form of *P*. *perniciosus* females, often misidentified as *P*. *longicuspis*, were counted as *P*. *perniciosus* [34,35]. Other sand fly species collected on the traps were identified to species level using the same aforementioned keys.

### Statistical analysis

To investigate sand fly preference for infected or uninfected dogs under laboratory conditions, we first compared numbers of female flies choosing side A or C by using a generalized linear model (GLM). A type II analysis of variance (ANOVA) was used to test for significant effects. The feeding success was analyzed using proportion data (females attracted / females feeding), because the input number of females in each cage was different. Feeding success was analysed with a GLM. Significant effects were tested with an ANOVA. Post-hoc analyses were performed with estimated marginal means (EMMEANS). Fecundity was analysed using a liner model and significant effects were determined with an ANOVA. Numbers of sand flies and eggs are given as mean ± SE. Similarly, proportions of fed sand flies (i.e. the number of females feeding divided by the number of females attracted) are given as mean ± SE.

To analyse the attractiveness of *P*. *perniciosus* to infected and uninfected dogs in the natural environment, a GLM with Poisson error was used to compare the number of sand flies between each treatment. Significant interactions and effects were tested using an ANOVA. Post-hoc analyses were performed using EMMEANS. The mean is the average number of specimens per variable in the different replicates. *P*-values less than 0.05 were considered to be significant and all analyses were performed using R v. 3.6.0.

## Results

### Attractiveness of *Phlebotomus perniciosus* towards infected and uninfected dogs under laboratory conditions

**Fig 3:**
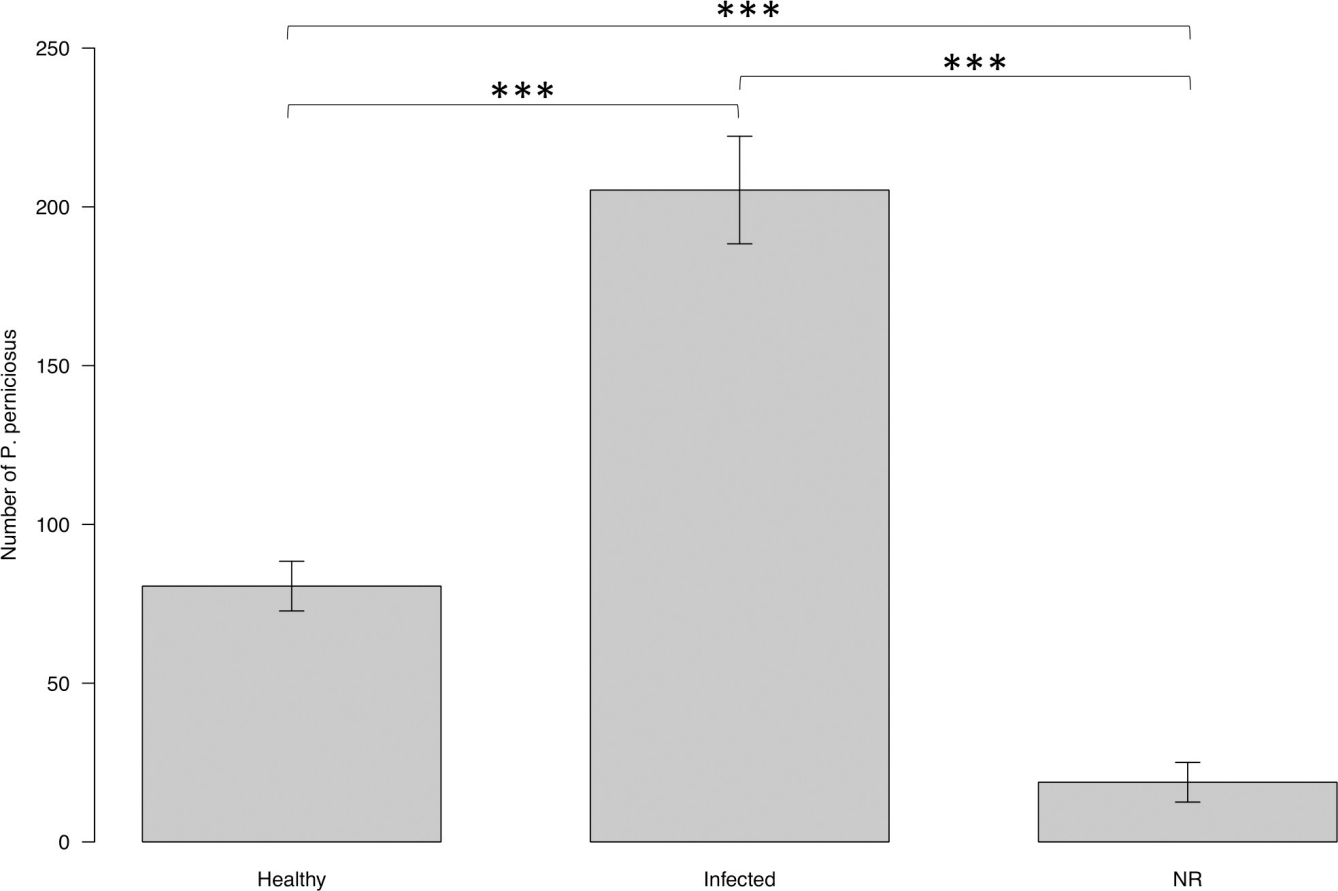
Mean number of females *P*. *perniciosus* attracted toward uninfected vs. infected dogs under laboratory conditions. Y axis represents the number of sand flies collected from the cage baited with infected and from the cage with uninfected dog. X axis represents the infection status of the dogs. Boxes are limited by the minimal and maximal value. Error bars correspond to the standard error. Significance code: p≤0.001 ***.

The numbers of female *P*. *perniciosus* attracted towards infected dogs and uninfected dogs were 205.30 ± 16.94 and 80.57 ± 7.82 respectively and 18.80 ± 6.25 did not respond (NR) (Fig 3). The attractiveness of the dogs differed significantly according to their infection status (ANOVA: F = 66.709, DF = 2, *P*<0.001) and infected dogs attracted significantly more *P*. *perniciosus* females than uninfected dogs (EMMEANS: z = 5.989, *P*<0.001, Fig 3).

**Fig 4:**
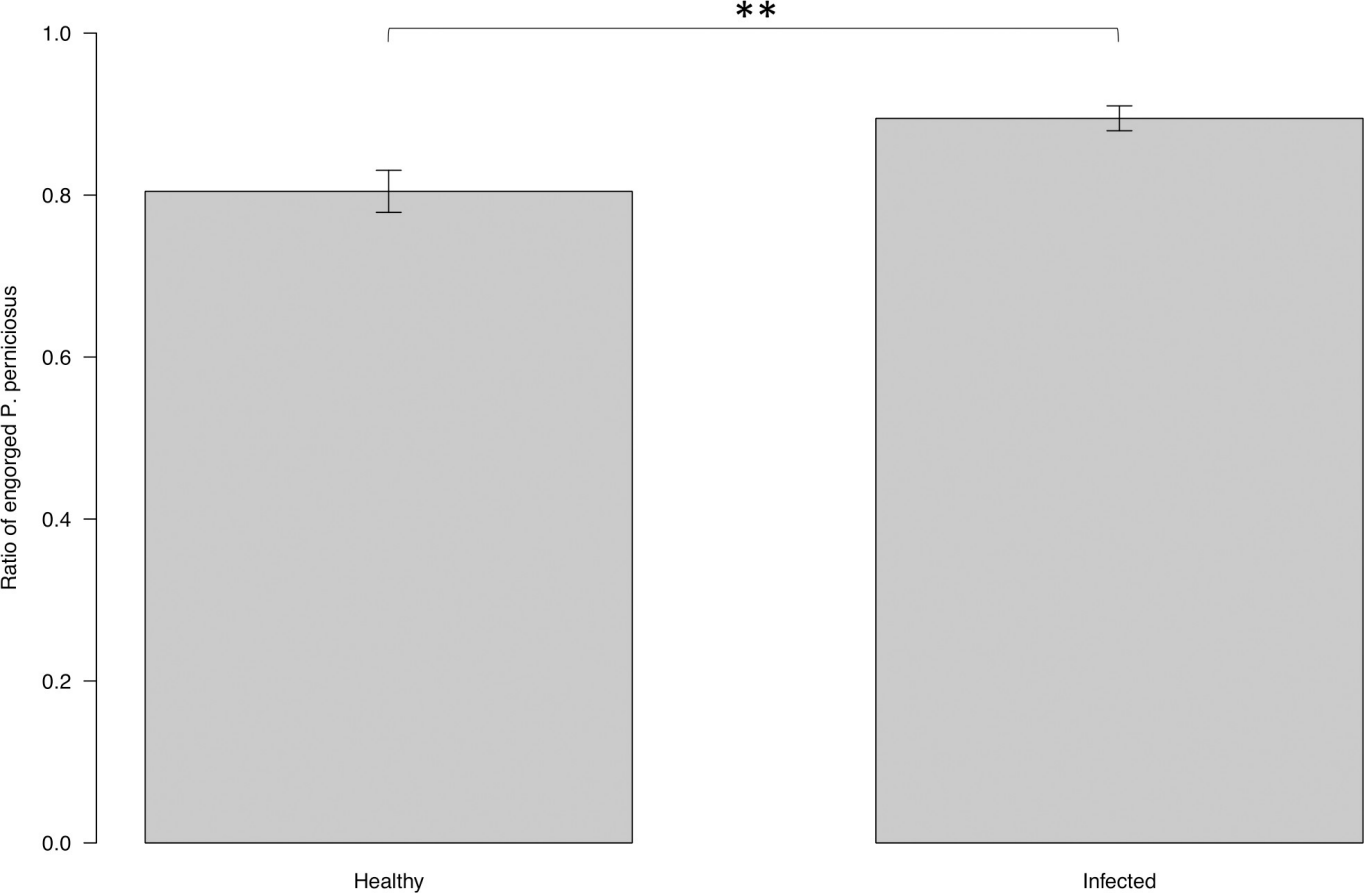
Proportion of females *P*. *perniciosus* fed on uninfected and on infected dogs under laboratory conditions. Y axis represents the proportion of engorged females collected from the cage baited with infected dog, and from the cage baited with uninfected dog. X axis represents the infection status of the dogs. Boxes are limited by the minimal and maximal value. Error bars correspond to the standard error. Significance code: p≤0.01 **.

The numbers of female *P*. *perniciosus* that fed on the infected and uninfected dogs were (186.16 ± 16.98), and (66.83 ± 7.58), respectively. The proportion of *P*. *perniciosus* females that fed on infected and uninfected dogs were 0.90 ± 0.02 and 0.81 ± 0.03 respectively (Fig 4). Feeding success was significantly affected by the infection status of dogs (ANOVA: F = 7.5714, DF = 1, *P* = 0.0079). The proportion of *P*. *perniciosus* females that fed on infected dogs was significantly higher than the proportion that fed on uninfected dogs (EMMEANS: z = 2.805, P = 0.005; Fig 4).

**Fig 5:**
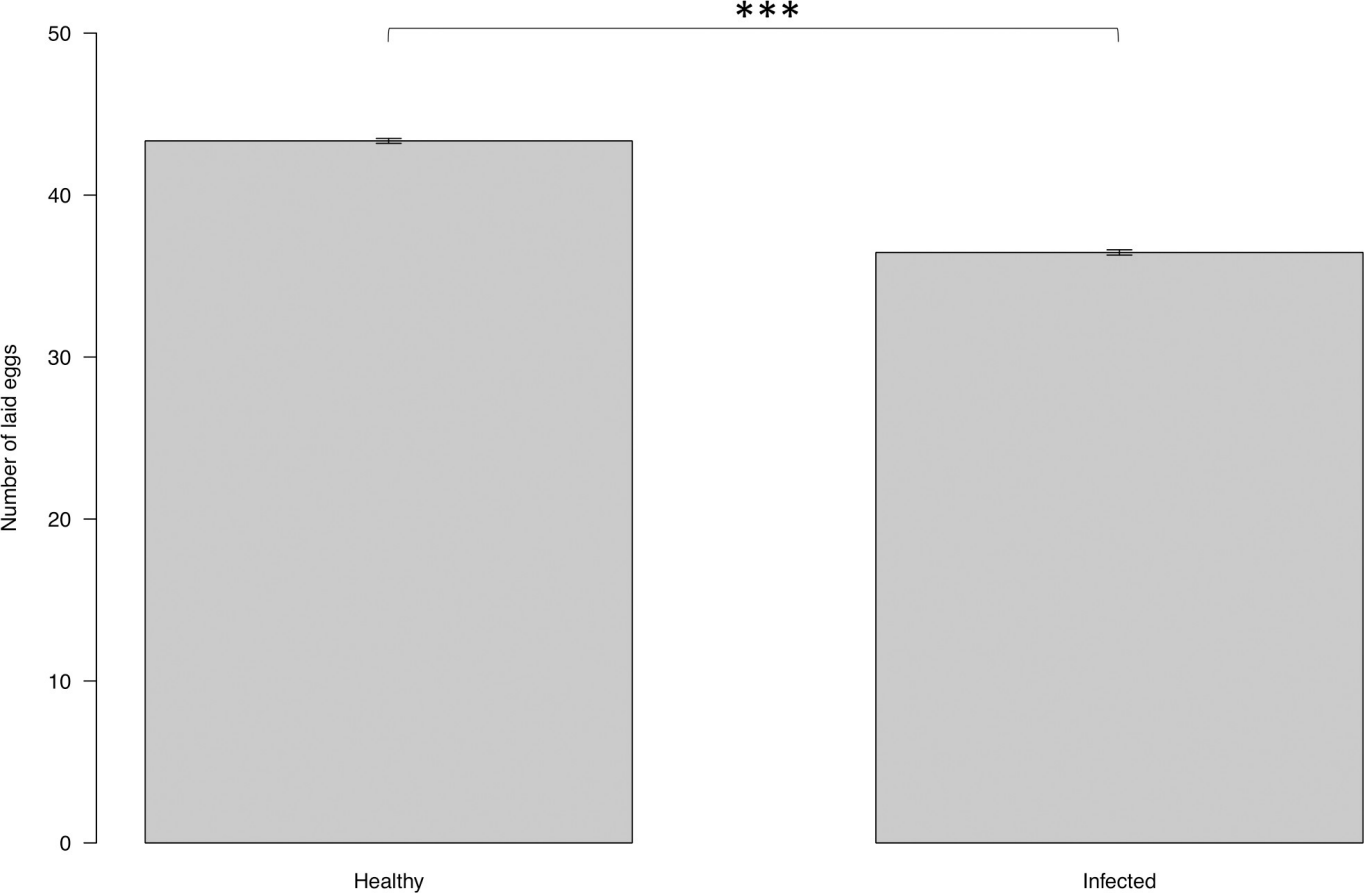
Mean number of eggs laid by *P*. *perniciosus* fed on uninfected vs. infected dogs. Y axis represents the numbers of eggs laid by *P*. *perniciosus* fed on infected and uninfected dogs X axis represents the infection status of the dogs. Significance code: p≤0.001 ***.

The number of eggs laid by each female *P*. *perniciosus* fed on infected dogs and uninfected dogs were 36.46 ± 0.16, and 43.35 ± 0.15, respectively (Fig 5). Fecundity was highly affected by the infection status of dogs (ANOVA: F = 13.35, DF = 1, P<0.001). Females that had fed on infected dogs laid significantly fewer eggs than females that had fed on uninfected dogs (EMMEANS: t = -3.654, P<0.001).

**Fig 6:**
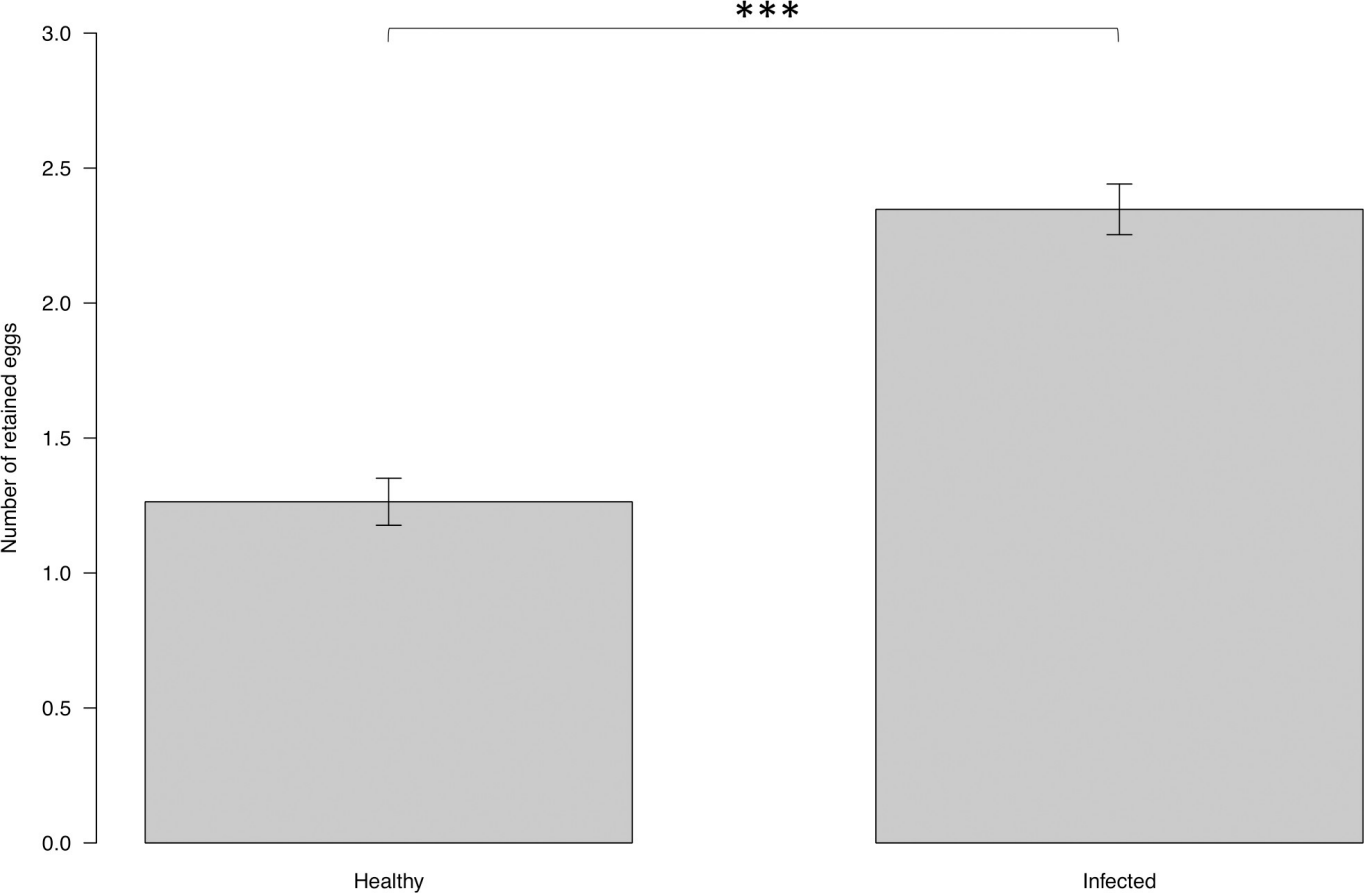
Mean number of retained eggs in *P*. *perniciosus* fed on uninfected vs. infected dogs under laboratory conditions. Y axis represents the numbers retained eggs in *P*. *perniciosus* fed on infected and uninfected dogs. X axis represents the infection status of the dogs. Significance code: p≤0.001 ***.

The number of retained eggs in females that had fed on infected dogs was 2.35 ± 0.09 and in females fed on uninfected dogs was 1.27 ± 0.09 (Fig 6). Similarly, the infection status of the host affected the number of retained eggs (ANOVA: F = 73.605, DF = 1, P<0.001).

### Attraction of sand flies to infected and uninfected dogs under field conditions

A total of 1,939 sand flies were collected during 27 trapping-nights. *Phlebotomus perniciosus* was the most abundant sand fly species (n = 1228, 63.3%) followed by *P*. *perfiliewi* (n = 662, 34.1%). The remaining sand fly species were *Phlebotomus papatasi* (n = 11, 0.56%), and *Sergentomyia minuta* (n = 38, 1.96%).

**Fig 7:**
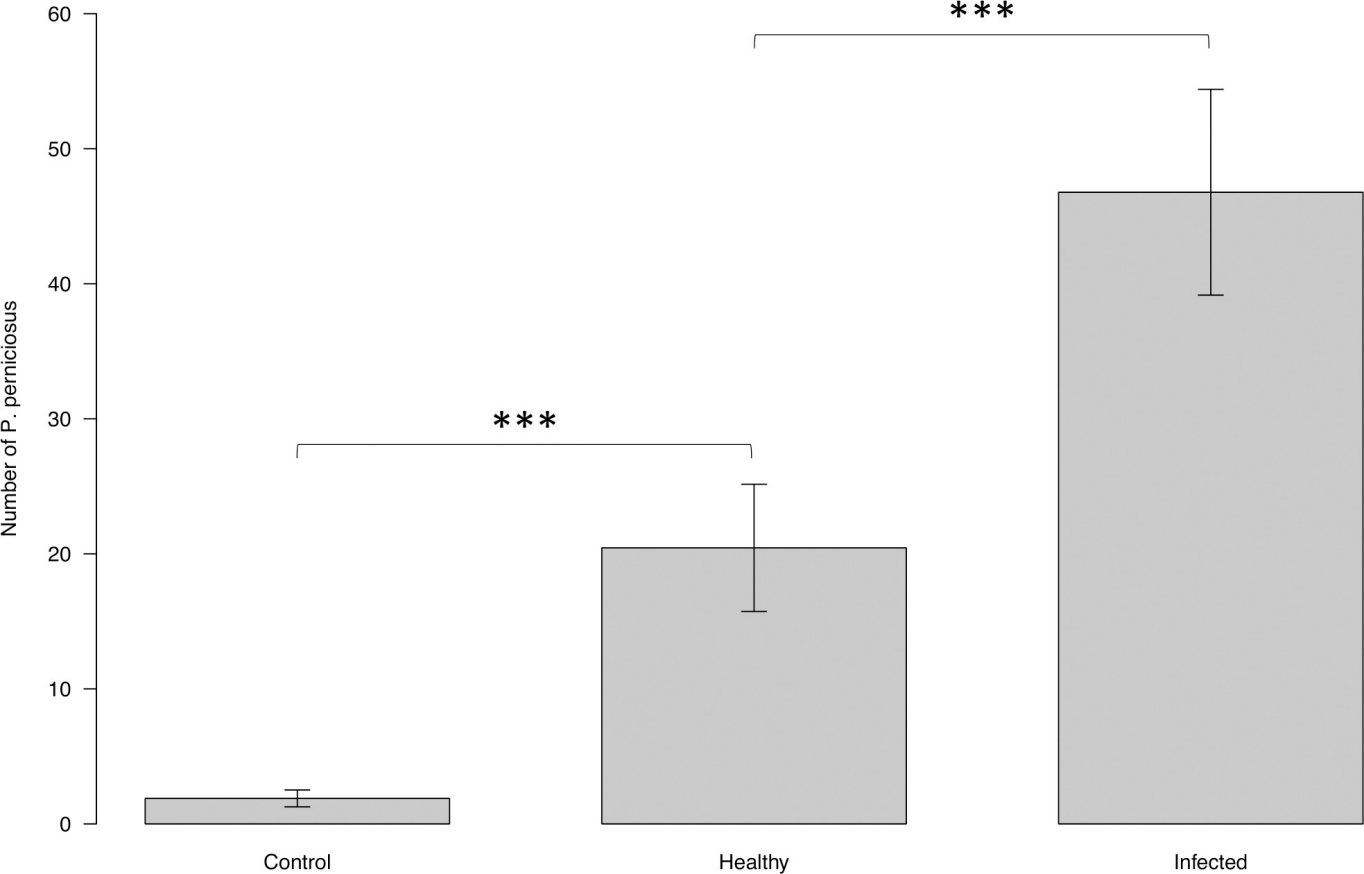
Mean number of *P*. *perniciosus* attracted toward uninfected vs. infected dogs under field conditions. Y axis represents the number of sand flies collected from the cage baited with infected dog, from the cage with uninfected dog, and from the un-baited cage. X axis represents the infection status of the dogs. Boxes are limited by the minimal and maximal value. Error bars correspond to the standard error. Significance code: p≤0.001 ***.

The numbers of *P*. *perniciosus* attracted to the infected dog, uninfected dog, and blank control were 20.44 ± 4.71, 46.78 ± 7.62, and 1.89 ± 0.63, respectively (Fig 7). Overall, there was a significant (ANOVA: F = 35.8120, DF = 2, *P* <0.001) difference in the numbers of *P*. *perniciosus* collected in the different cages. Infected dog attracted more *P*. *perniciosus* than uninfected dog (MCP: z = 3.666, *P*<0.001). Both infected and uninfected dogs attracted more *P*. *perniciosus* than empty cage (MCP: uninfected: z = 3.679, *P*<0.001; infected: z = 5.079, *P*<0.001).

**Fig 8:**
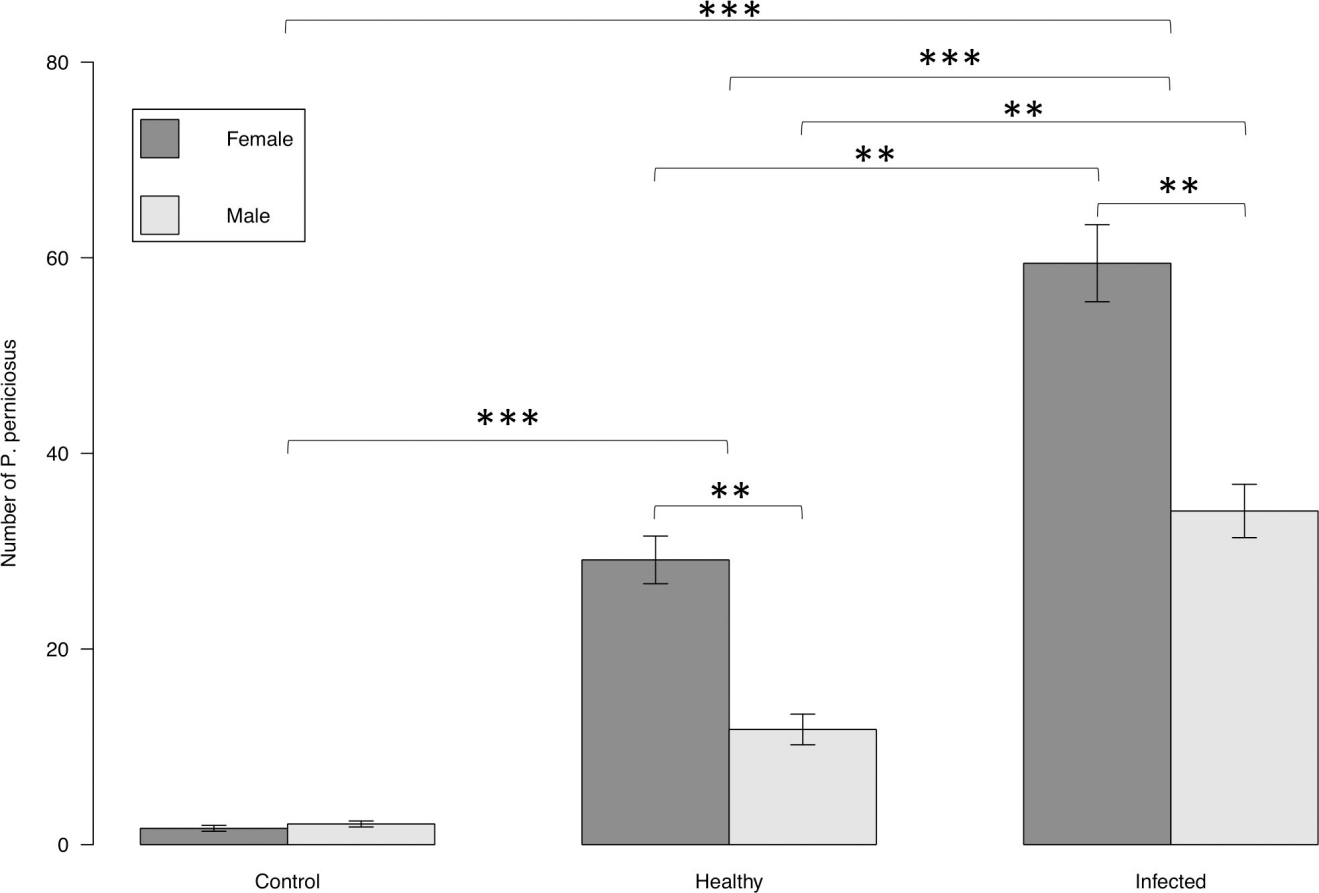
Mean number of males and females *P*. *perniciosus* attracted toward uninfected vs. infected dogs under field conditions. Y axis represents the number of sand flies collected from the cage baited with infected dog, from the cage with uninfected dog, and from the un-baited cage. X axis represents the infection status of the dogs. Boxes are limited by the minimal and maximal value. Error bars correspond to the standard error. Significance code: p≤0.01 **; p≤0.001 ***.

The number of females trapped was significantly higher (30.07 ± 6.43) than the number of males (16.00 ± 4.02) (MCP: z = 2.933, *P* = 0.00335) (Fig 8). The number of female *P*. *perniciosus* trapped in cages baited with the infected dog (59.44 ± 11.82) was significantly greater than the number of males trapped (34.11 ± 8.18) (EMMEANS: z = 2.933, *P* = 0.0393). Similarly, the number of female *P*. *perniciosus* trapped in the cage baited with the uninfected dog (29.11 ± 7.31) was significantly greater than the number of males trapped (11.78 ± 4.70) (EMMEANS: z = 2.933, *P* = 0.0393). The number of male *P*. *perniciosus* trapped in cages baited with the infected dog (34.11±8.18) was significantly greater than the mean number of males trapped in the cage baited with the uninfected dog (11.78 + 4.70) (EMMEANS: z = 3.666, *P* = 0.0034) (Fig 8). Similarly, the mean number of females *P*. *perniciosus* trapped in the cage baited with the infected dog (59.44 + 11.82) was significantly higher than the mean number of females trapped in cages baited with the uninfected dog (29.11 + 7.31) (EMMEANS: z = 3.666, *P* = 0.0034).

**Fig 9:**
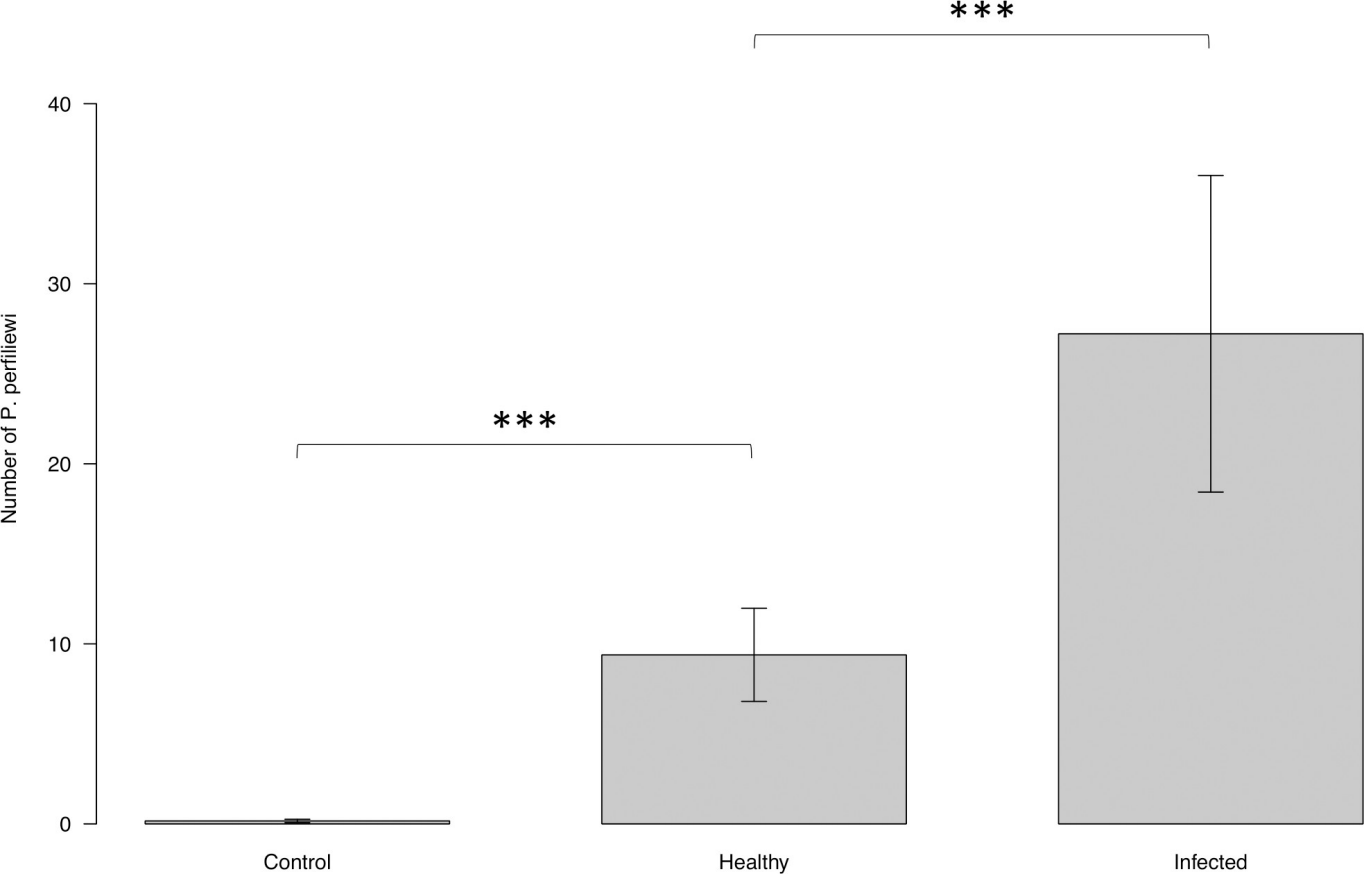
Mean number of *P*. *perfiliewi* attracted toward uninfected vs. infected dogs under field conditions. Y axis represents the number of sand flies collected from the cage baited with infected dog, from the cage with uninfected dog, and from the un-baited cage. X axis represents the infection status of the dogs. Boxes are limited by the minimal and maximal value. Error bars correspond to the standard error. Significance code: p≤0.001 ***.

The numbers of *P*. *perfiliewi* attracted towards the infected dog, uninfected dog, and empty control cage were 27.22 ± 8.79, 9.39 ± 2.59 and 0.17 ± 0.09, respectively (Fig 9). Overall, the difference between numbers of *P*. *perifiliewi* collected in the cages with the infected dog, uninfected dog, and empty control cage was statistically significant (ANOVA: F = 94.715, DF = 2, *P* <0.001). Infected dog attracted more *P*. *perfiliewi* than uninfected dog (EMMEANS: z = 7.931, p<0.001). Both infected and uninfected dogs attracted more *P*. *perfiliewi* than empty control cage (EMMEANS: infected: z = 13.440, *P*<0.001; uninfected: z = 9.005, *P*<0.001).

Overall, the numbers of male and female *P*. *perfiliewi* trapped were 4.59± 1.49 and 19.53±6.29, respectively. The mean number of females trapped was significantly higher than the number of males (EMMEANS: z = 10.52, *P*<0.001). The number of female *P*. *perfiliewi* trapped in the cage baited with infected dog (44.22 ± 15.56) was significantly greater than the number of males trapped (10.22 ± 3.79) (EMMEANS: z = 10.425, *P*<0.001). Similarly, the mean number (mean ± SE) of female *P*. *perfiliewi* trapped in the cage baited with the uninfected dog (15.44 ± 4.30) was significantly greater than the number of males trapped (3.33 ± 0.88) (EMMEANS: z = 7.344, *P*<0.001). The number of male *P*. *perfiliewi* trapped in the cage baited with the infected dog was significantly greater than the mean number of males trapped in the cage baited with the uninfected dog (EMMEANS: z = 3.990, *P* = 0.0013). Similarly, the mean number (mean ± SE) of female *P*. *perfiliewi* trapped in the cage baited with the infected dog was significantly higher than the mean number of females trapped in the cage baited with the uninfected dog (EMMEANS: z = 9.260, *P*<0.001).

## Discussion

In this study we investigated the effect of infection with *L*. *infantum* on the attractiveness of dogs in both the laboratory and the field in a natural focus of transmission. We tested the attractiveness of six pairs of dogs, matched for sex and age, one dog was infected (and symptomatic) and the other uninfected in both studies. Our results showed that dogs infected with *L*. *infantum* were highly attractive to both female and male *P*. *perniciosus*. The sand flies had a choice of orientating towards either of the potential hosts, but in all six laboratory replicates (pairs of dogs) female *P*. *perniciosus* were significantly more attracted to the infected than uninfected dogs. Similarly, we observed the same response pattern when one of the pair of dogs was exposed to the wild population of *P*. *perniciosus*.

The feeding success of *P*. *perniciosus* is significantly higher on infected dogs compared to uninfected ones. However, *P*. *perniciosus* that had fed on the uninfected dogs laid significantly more eggs than those that fed on dogs infected with *L*. *infantum*. Similar results were observed when *P*. *langeroni* and *L*. *longipalpis* were fed artificially on *L*. *infantum* infected blood [36,37]. While the longevity of *L*. *longipalpis* was reduced after artificial feeding on *L*. *infantum* infected blood, no significant impact on the fecundity was reported [38]. All the aforementioned studies were obtained from artificially infected sand flies. Since we used a natural parasite-vector-host system, our results strongly suggest that *L*. *infantum* infection exerted an adverse impact on the fecundity of *P*. *perniciosus*. Resources for egg production may be diverted to limit the reduced longevity following infection with *L*. *infantum*. This hypothesis deserves further investigation.

Our study did not fully determine if the enhanced attractiveness of infected dogs that we observed was the result of host odour, thermal, visual or acoustic (or a combination of some or all of them) cues. However it is very likely that the sand flies are responding predominantly to odour cues. Several studies have suggested that dog odours are altered by *L*. *infantum* infection. Magalhaes et al. (2014) [39] identified 35 volatile organic components (VOCs) emitted by infected dogs that were either quantitatively or qualitatively different to uninfected dogs and could be recognised as bio-markers of infection. Similarly, a study using a VOC analyser (eNose) showed that dogs naturally infected with *L*. *infantum* in Brazil had a significantly different odour profile when compared to uninfected dogs [40].

Several other studies on non-natural infection systems that have also shown that odour of animals infected with *L*. *infantum* plays an important role in increasing the attractiveness of the infected animal. Nevatte et al. (2017) [24] showed that the attractiveness of golden hamsters (which do not naturally become infected with *L*. *infantum*) to *L*. *longipalpis* increased significantly after infection. The change in attractiveness was related to stage of infection and the change was more pronounced in some individuals than others [24].

Similar results have been obtained with other vector-parasite-host systems. Infection of the mouse with the parasite *Plasmodium chabaudii*, the etiological agent of rodent malaria, induces an increased attractiveness of *Anopheles stephensi* [41]. A clear difference in the VOCs of mice infected with *P*. *chabaudii* compared to those of uninfected mice has been shown [41]. Likewise, humans infected with *P*. *falciparum* are more attractive to *Anopheles gambiae* [42]. However, there is evidence that the effect may not be universal e.g. there was no significant difference in the attractiveness of the sand fly *Nyssomyia neivei* toward BALB/c mice infected with *Leishmania braziliensis* and uninfected mice [43].

In order to confirm our laboratory results, we tested the attractiveness of dogs infected with *L*. *infantum* under natural conditions in a highly endemic focus for canine leishmaniasis. Our results showed that the number of *P*. *perniciosus* collected from the cage housing symptomatic *L*. *infantum* infected dog was significantly higher than the number of flies collected from the cage baited with uninfected dog. We observed the same pattern of responses from the wild population of *P*. *perfiliewi* to the same pair of dogs. This evidence strongly suggests that infected dogs are more attractive to both species of sand flies. The numbers of non *L*. *infantum* vectors species attracted to the dogs in the field experiments was too low to determine if there was a preference for infected rather than uninfected dogs. Our manipulation hypothesis would predict that there would be no difference in attraction of non-vector species to infected and uninfected dogs.

The enhanced attractiveness of *P*. *perniciosus* and *P*. *perfiliewi* to dogs infected with *L*. *infantum* under natural conditions is most likely due to the difference in the kairomones produced by the infected and uninfected dogs. A consequence of this manipulation could be enhanced transmission success of the parasite *L*. *infantum* to the vector. It would be interesting in the future to determine when the enhanced transmission success occurs in relation to the parasite life cycle in the host.

Our study showed that although both females and males *P*. *perniciosus* were attracted to uninfected dogs they were significantly more attracted to infected dogs in both the lab and field-based study. The response of the males seen here contrasts with the response of male *L*. *longipalpis* seen in other studies where there was no increased attraction of males to infected hamster [24]. This study overcomes the limitations of the previously reported work, as it was partly carried out in the field with the presence of wild sand flies in an endemic ZVL focus and thus is the closest representation of natural transmission.

Based on blood meal analysis *P*. *perniciosus* is seen to be opportunistic feeding on whichever hosts are available [21,44–47]. No field studies on *P*. *perniciosus* have been carried out to investigate their relative attractiveness to different hosts. There is some evidence that *L*. *longipalpis* chooses hosts on the basis of their relative size rather than species [48], it is generally also considered to be an opportunistic feeder. The general zoophilic feeding behaviour of *P*. *perniciosus* and *L*. *longipalpis* based on host availability rather than on attractiveness to specific hosts should lead to a dilution of the parasite as it is spread to non-competent hosts, and this would subsequently exert a zooprophylactic effect on the transmission of *L*. *infantum* [49]. Our work illustrates a potential zoopotentiation effect exerted by infected reservoir hosts that are highly attractive to sand fly vectors of *L*. *infantum*. Furthermore, it strongly suggests that infected dogs are the main reservoir host, as the different odour components and/or different concentrations that are released are significantly attractive to *P*. *perniciosus* compared to other environmental odours. This odour manipulation ensures successful transmission of the parasite to the vector. Thus, chemical ecology governs the *L*. *infantum*-dog-*P*. *perniciosus* relationship, and consequently, it has a direct impact on the transmission dynamic of ZVL.

While it has been shown that *Leishmania* infection may influence the quantity of blood ingested and the frequency of sand fly blood meals, thereby increasing the transmission rate of the parasite [38,50], it is not known whether parasites can affect host attractiveness to sand flies. Some parasites are known to manipulate their host animals by changing its physiology or behaviour to improve their chance of transmission [22]. Our results strongly suggest that the parasite changes the physiology of the dog so that it becomes more attractive to female *P*. *perniciosus*, thus helping to ensuring its successful transmission.

Several studies have reported that symptomatic dogs, infected with *L*. *infantum* are highly infectious to their sand fly vectors compared to oligosymptomatic and asymptomatic dogs [51–58]. One explanation for this difference may be related to the relative attractiveness of symptomatic infected and uninfected dogs to their sand fly vectors. It will be interesting in due course to determine if infected asymptomatic and oligosympotomatic dogs are as attractive to the sand fly vectors as symptomatic dogs.

Greater attractiveness of infected dogs compared to uninfected dogs would have major epidemiological significance. In ZVL endemic areas of Tunisia, up to 50% of dogs are infected with *L*. *infantum* [4], therefore understanding the mechanisms which underpin the difference in attractiveness could help in the development of new approaches to reduce the infection rate of the vector, and subsequently to reduce the transmission of the parasite. It is also important to understand how the parasite may manipulate transmission and thus the effect on transmission models.
