# Correction: Enhanced attraction of sand fly vectors of *Leishmania infantum* to dogs infected with zoonotic visceral leishmaniasis

**DOI:** 10.1371/journal.pntd.0009947

**Published:** 2021-11-11

**Authors:** Ifhem Chelbi, Khouloud Maghraoui, Sami Zhioua, Saifedine Cherni, Imen Labidi, Abhay Satoskar, James G. C. Hamilton, Elyes Zhioua

There are errors in the Funding statement. The correct Funding statement is as follows: The authors received no specific funding for this work.

The US National Institutes of Health and the Wellcome Trust did not provide any funding for this research and any such claim was made in error.

There is an error in the Ethics statement. The correct Ethics statement is as follows: The maintenance of animals and the experimental procedures used in this research program followed the Animal Care and Use Protocol which is approved by the Institutional Animal Care and Use Committee of the Institut Pasteur de Tunis, Tunisia (2018/01/I/ES/IPT/V0). Infected dogs used in this research program were obtained from a previous industry sponsored study that was approved by the Institutional Animal Care and Use Committee of the Institut Pasteur de Tunis, Tunisia (IPT/UESV/27/2012). The Institut Pasteur de Tunis complies with the European Directive for the Protection of Vertebrate Animals used for experimental and other scientific purposes (2010/63/EU).
